# Splicing mutations in the *CFTR* gene as therapeutic targets

**DOI:** 10.1038/s41434-022-00347-0

**Published:** 2022-06-02

**Authors:** Karine Deletang, Magali Taulan-Cadars

**Affiliations:** 1 PhyMedExp, Université de Montpellier, INSERM U1046, CNRS UMR, 9214 Montpellier, France; 2 Université de Montpellier, Montpellier, France

**Keywords:** Gene regulation, RNA metabolism

## Abstract

The marketing approval, about ten years ago, of the first disease modulator for patients with cystic fibrosis harboring specific CFTR genotypes (~5% of all patients) brought new hope for their treatment. To date, several therapeutic strategies have been approved and the number of CFTR mutations targeted by therapeutic agents is increasing. Although these drugs do not reverse the existing disease, they help to increase the median life expectancy. However, on the basis of their CFTR genotype, ~10% of patients presently do not qualify for any of the currently available CFTR modulator therapies, particularly patients with splicing mutations (~12% of the reported CFTR mutations). Efforts are currently made to develop therapeutic agents that target disease-causing CFTR variants that affect splicing. This highlights the need to fully identify them by scanning non-coding regions and systematically determine their functional consequences. In this review, we present some examples of CFTR alterations that affect splicing events and the different therapeutic options that are currently developed and tested for splice switching.

## Introduction

**Fig. 1:**
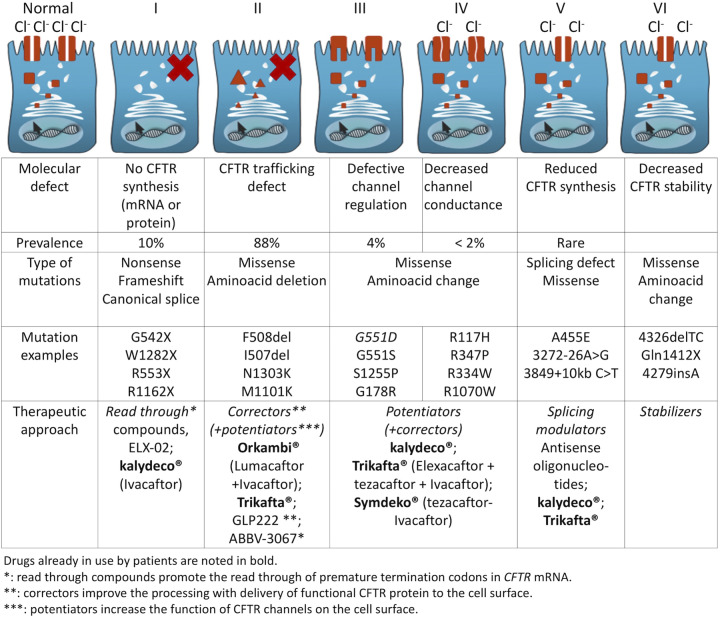
Molecular defect of CFTR mutations and therapeutic approaches developed.

The *CFTR* (Cystic Fibrosis Transmembrane conductance Regulator) gene displays great mutational heterogeneity, depending on the ethnic and genetic background. Today, more than 2000 CFTR variants have been identified in patients with Cystic Fibrosis (CF) and CFTR-related disorders. Some of these variants are known to cause CF, but many are still uncharacterized and named as variants of unknown significance. CFTR disease-causing mutations have been classified in six classes (I to VI) depending on their effect on protein production, maturation, folding, activity, conductance, and stability at the cell surface [1, 2] (Fig. 1). Some mutations belong to two or more classes [3].

In 2012, ivacaftor (VX-770) was the first molecule approved by the US Food and Drug Administration (FDA) for CF treatment. Ivacaftor is a potentiator therapy because it binds to the defective CFTR protein at the membrane surface and helps to keep chloride channels open. It was initially approved only for patients with the CFTR G551D mutation, the third most common mutation (the G551D mutation, at least on one allele, is detected in ~4–5% of patients with CF) [4], but has now be authorized for 97 CFTR mutations with lower prevalence. In 2015, the combination (Orkambi®) of a corrector (Lumacaftor, VX-809) and a potentiator (Ivacaftor) was approved for ≥12-year-old patients with two copies of the p.Phe508del (c.1521_1523del) CFTR mutation (~44% of the CF population). Since 2018, its approval has been extended to ≥2-year-old patients with two copies of the p.Phe508del mutation. In 2019, the FDA approved Trikafta®, a triple-combination therapy (Elexacaftor [Vx445] / Tezacaftor [VX-661] / Ivacaftor [VX-770]) for ≥12-year-old patients with CF harboring at least one copy of the p.Phe508del mutation. In 2021, Trikafta® was authorized also for ≥6-year-old patients with at least one copy of the p.Phe508del mutation and other disease-causing-variants. Clinical trials with other drugs are under way. The combination of Tezacaftor and VX-121 (two potentiators that should increase the amount of mature CFTR at the cell surface) and VX-561 (a modified form of VX-770 that should increase CFTR function) is tested in a phase 3 clinical trial. New modulators are currently evaluated in phase 2 trials, such as GLPG2222 (a corrector of CFTR function) and ABBV-3067 (a potentiator assessed in patients with CF harboring two copies of p.Phe508del) [5]. For patients harboring nonsense mutations, which generally yield no functional CFTR, a novel small compound, ELX-02 (known also as NB124), has been developed. This molecule promotes read-through of mRNA transcripts bearing nonsense mutations, including the most common CF nonsense allele (G542X), to override these premature stop signals and produce functional full-length CFTR protein [6]. ELX-02 is well tolerated and its pharmacokinetic properties are consistent with the preclinical data [7]. On-going clinical trials evaluate ELX-02 as a read-through agent in patients with CF caused by the G542X allele.

CFTR modulator therapy has revolutionized the care of patients with CF and also with other diseases, for instance sarcoglycanopathies [8]. However, the majority of patients with CF harboring mutations affecting splicing events (i.e., splicing mutations), are not eligible to receive these highly effective CF modulators (with some exceptions, see Supplementary Tables 1 and 2). This review discusses the existing alternative strategies for these patients. Splicing mutations represent ~12% of all reported CFTR mutations. More than 200 splicing variants are listed in the Cystic Fibrosis Mutation Database (www.genet.sickkids.on.ca), a research database run by researchers at the Hospital for Sick Children in Toronto, Ontario, Canada. The most frequent splicing variants referenced in the CFTR2 database that contains data on 88,000 patients with specific CF variants from the United States, Canada, and Europe (https://cftr2.org) are listed in Supplementary Table 1. Most of them affect the canonical AG (3’splicing site) and GU (5’splicing site) motifs. Others (1–2% of CFTR mutations) remain undetected and are probably located deep in introns where they induce aberrant splicing events. The development of next-generation sequencing (NGS) technologies with an immense capacity (up to 600 Gb per run) represents a major progress in human genetics. Whole-genome and exome sequencing are becoming popular approaches, and exome sequencing can be a powerful tool to identify the molecular basis of single-gene disorders [9]. The main advantage of this technology is the possibility to completely sequence a gene, including introns. We and others used NGS to scan the whole *CFTR* gene, including the non-coding regions, to identify new intronic mutations [10–12]. Nevertheless, the number of rare sequence alterations in introns is probably underestimated, and NGS technologies will extend the catalog of new intron variants.

Here, we present the mutations that affect the normal splicing events of the *CFTR* gene and an overview of the drugs and therapeutic strategies that have been developed to target these mutations.

## Splicing mutations of the *CFTR* gene

Analysis of genome-wide sequencing data, including mRNA/EST data, revealed that 60% of mammalian genes are alternatively spliced in various patterns [13, 14]. Classical alternative splice events include exon skipping/inclusion (cassette exons), alternative 5’ or 3’ splice sites, mutually exclusive exon use, intron retention, and various combinations.

**Fig. 2:**
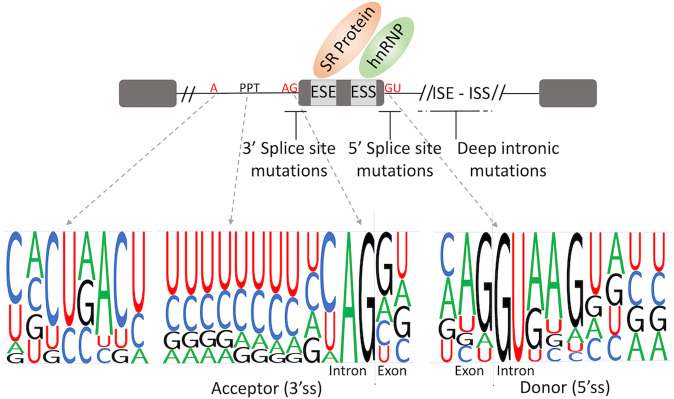
Key splicing motifs and splicing regulatory elements. Gray boxes represent exons, full black lines represent introns, ESE Exonic Splicing Enhancer, ESS Exonic Splicing Silencer, ISE Intronic Splicing Enhancer, ISS Intronic Splicing Silencer, 3’SS 3’ Splicing Site, 5’SS 5’ Splicing Site.

The splicing mechanism comprises exon recognition in the pre-mRNA transcript and the removal of flanking introns (Fig. 2). The core splicing signals are the intronic branch point sequence, the acceptor site (3’splicing site), with a variable upstream polypyrimidine tract, and the donor site (5’ splicing site). These core splice site motifs contain only part of the information needed for exon definition. The remaining information is in less conserved regions that are called splicing regulatory elements (SRE) and are located in the exon or flanking introns. They promote and inhibit exon recognition through exonic/intronic splicing enhancers (ESE or ISE) and silencers (ESS or ISS), respectively.

### Mutations that affect the canonical acceptor and donor sites

Mutations affecting splicing events may occur in introns and exons. They may disrupt existing splice sites or splicing regulatory sequences (intronic and exonic splicing silencers and enhancers), create new ones, such as acceptor and donor sites, or activate cryptic ones, and induce the incorporation of pseudo-exons [15]. If the open reading frame is disrupted, a premature termination codon (PTC) can arise, and a shorter protein can be produced. However, PTC-containing transcripts may be degraded by the nonsense-mediated mRNA decay (NMD) pathway [16]. NMD is a post-transcriptional RNA surveillance mechanism that is activated to control the quantity of PTC-containing transcripts generated as a consequence of gene mutations or inefficient/inaccurate splicing. Human studies have shown that ~92–94% of genes are alternatively spliced [17], and that 40% of alternative splicing events can generate in-frame PTCs, which are natural targets of the NMD pathway. The degradation of PTC-containing transcripts is important to prevent the production of truncated proteins that can have a dominant-negative effect.

In mammals, about 55% and 45% of splicing mutations affects the canonical GT and AG dinucleotides, respectively, that create the consensus sequences of donor/acceptor splice sites [15]. The most classical splicing mutations affect +1 and +2 residues at the 5′ donor and −1 and −2 residues at the 3′ acceptor splice site. A meta-analysis of splicing mutations (478 mutations in 38 selected genes) [15] showed that donor splice site mutations are more frequent than acceptor splice site variants (ratio 1.5:1) (Ref. [18]).

Among the CFTR splicing mutations referenced in the Cystic Fibrosis Mutation Database (the most frequent are listed in Supplementary Table 1, CFTR2 database), more than 200 correspond to splicing mutations in the canonical acceptor and donor sites. CFTR splicing mutations that alter canonical splice sites are in class I and mutations that create alternative splice sites are in class V. Class I mutations lead to defective protein synthesis due to nonsense mutations (class Ia) or splicing disruption (class Ib). Class V mutations lead to very low levels of functional protein due to reduction of normal *CFTR* transcripts caused by the production of aberrantly spliced transcripts [19].

Usually, mutations at canonical splice sites result in single exon skipping. Nevertheless, an alternative splice site can be used during splicing if the normal splice site is weak or if the mutation creates or activates a cryptic splice site. This can lead to the inclusion of an intron fragment if the cryptic splice site is located in an intron, or to the removal of an exon fragment if the cryptic splice site is in an exon. For example, for the c.1393−1G>A mutation (legacy name 1525−1G>A) in intron 10 of the *CFTR* gene, three mRNA isoforms have been characterized: one lacking whole exon 10 and two harboring only fragments of exon 10. The authors identified distinct alternative splice sites located in intron 10 and exon 10 (positions c.1610–1611 and c.1678–1679) for the last two isoforms [20]. In the CFTR-France Database that currently includes 5267 individuals among whom 3135 patients with CF from ten French laboratories (https://cftr.iurc.montp.inserm.fr/cgi-bin/home.cgi), few splicing mutations are among the 40 more frequent CFTR mutations in France (>0.3%): c.2657+5G>A (2789+5G>A), c.1585−1G>A (1717−1G>A), c.579+1G>T (711+1G>T), c.2988+1G>A (3120+1G>A), c.489+1G>T (621+1G>T), and c.3873+1G>A (4005+1G>A). They are all considered CF-causing mutations in the CFTR2 database (https://cftr2.org). Although the c.2657+5G>A mutation shows a very high variability, 60% of patients with CF harboring this mutation in the CFTR2 database have normal pancreatic function. Moreover, the c.2657+5G>A mutation has been detected in several patients with CFTR-related disorders (such as congenital bilateral absence of vas deferens (CBAVD), bronchiectasis, or pancreatitis) carrying a CF-causing mutation in *trans* (CFTR-France database). The 5T allele, located in a Tn polymorphic tract in intron 9, is another well-described splicing variant that promotes *CFTR* exon 10 skipping. In the general population, three major alleles are found at the Tn locus: 5T (5% of all alleles), 7T (84%), and 9T (11%) (Ref. [21]). The 7T and 9T alleles are considered non-pathogenic. Conversely, the 5T allele is associated with extreme variability in disease expression, ranging from healthy fertile individuals or males with CBAVD to individuals with typical severe CF pulmonary disease [22]. The phenotype variability associated to the 5T allele is related to the associated TG repeat: from mild or late onset CF for TG13T5 to almost absence of symptoms for the TG11T5 combination [23, 24]. These studies highlighted that variability in disease expression correlates with the amount of correctly spliced *CFTR* mRNA [22].

### Exon mutations that affect splicing events

Some exon mutations (e.g., missense, nonsense, small deletions) can affect splicing events [25–30]. Missense mutations can influence exon inclusion and nonsense mutations can disrupt exon choice by altering the NAGNAG motif [31]. NAGNAG acceptor motifs are present in 30% of human genes, and can lead to alternative splicing and transcripts. The use of the intron proximal or distal acceptor splice site results in the production of two distinct isoforms distinguished by three nucleotides (NAG) that create or delete a PTC. Several studies [31, 32] have shown that *CFTR* non-sense mutations, such as the p.Glu831* (E831X) mutation (c.2491G>T) (ref. [31]), generate a PTC on the NAGNAG acceptor site (GAG changed to TAG). Two different mechanisms can explain how nonsense mutations (e.g., p.Arg553* (R553X), c.1657C>T), preferentially cause exon skipping compared with other mutation types [26]. The first mechanism is nonsense-associated altered splicing that might involve a scanning process to monitor the open reading frame for PTCs before splicing, thus promoting skipping of the exon in which the PTC is located. Nonsense-associated altered splicing was described as a mechanism to protect PTC-containing transcripts from NMD [33]. During this process, PTC-containing transcripts are alternatively spliced and fragments with the premature nonsense codon are removed from the mature RNA. The second mechanism, which seems more obvious, involves the inactivation of ESEs, which are short and degenerate sequences that promote the selection of proximal splice sites in constitutive and alternative exons [26].

Exon mutations can also create a new 5’ or 3’ splice site, or favor the use of a cryptic site, stronger than the canonical site [15]. These events lead to pre-mRNA processing alterations and loss of exon fragments, eventually producing a PTC-containing immature protein. In exons, SRE presence also can be affected by mutations, resulting in altered recruitment of trans-regulatory elements, such as ESE and ESS motifs [25–27,29]. These SREs recruit SR family splicing factors, for example, to activate splice site recognition. Some CFTR variants initially considered to be neutral (c.220C>T, c.224G>A) can disrupt ESE motifs and cause exon 3 skipping [27]. Specifically, these mutations weaken the functional ESE motif in *CFTR* exon 3 that recruits the SR protein SF2/ASF [34].

### Deep intronic mutations and splicing defects

The NGS technology allows scanning of the whole *CFTR* gene, including introns [10–12,35]. Analysis of intron sequences revealed another category of splicing mutations: intronic mutations the frequency of which is probably underestimated for different reasons. These alterations are located deeply in introns. These regions are not routinely sequenced, and in silico tools to predict putative splicing defects in introns remain limited and challenging. Indeed, although the number of in silico tools to predict splicing defects has been increasing, they usually can predict the consequence only of variants affecting the 5’ and 3’ splice sites. These tools are based on the calculation of the ESS/ESE ratio (Skippy [36], EX-SKIP [37], ΔtESRseq [38]), the ΔHZ_EI_ score between mutant and reference sequences (HEXplorer [39]), variation in splicing rate (Δψ, Spidex [40]), and machine learning algorithms to identify splicing variants (MutPred Splice [41], Splice AI [42]). The impact of variants located in branch points, poly-pyrimidine tracts, SREs, and far from exons (e.g., deep intronic mutations) is harder to predict. Currently, the putative impact of deep intronic mutations needs to be manually investigated. Indeed, if the prediction tool suggests that a variant creates or reinforces the score for non-canonical donor or acceptor sites (to learn more about in silico algorithms for splicing variant impact prediction, see ref. [15]), the presence of pre-existing non-canonical strong donor or acceptor sites at a distance of 50–3000 bp is required to consider a possible defect. These variants, usually located within introns, result in the inclusion of an intron sequence, called cryptic exon or pseudo-exon, into the mature transcript. If the insertion of the intronic sequence creates a shift in the reading frame, it generally leads to the appearance of a PTC during translation. In these cases, mRNA analysis could be proposed as a routine procedure to directly identify and characterize these splicing variants; however, various factors still limit its use, particularly, sample accessibility, low quantity and stability, and the NMD-dependent degradation rate of the PTC-containing mRNA.

In addition, considerable variability in the level of endogenous correctly and aberrantly spliced *CFTR* mRNA is found in samples from different CF patients with deep intronic mutation (listed in Supplementary Table 2 [12, 22,43–52]). Phenotypic variability has been largely reported for c.3718−2477C>T (3849+10kbC>T) (ref. [53, 54]) and this mutation showed a correlation between the amount of aberrant splicing and disease severity [55]. Patients with the genotype c.1521_1523del;c.1680−886A>G (F508del;1811+1.6kbA>G) have a very low level of functional mRNA (1–3%), explaining the pancreatic insufficiency and their severe CF phenotype [46]. The clinical features of patients homozygous for the c.1680−886A>G (1811+1.6kbA>G) showed that the phenotype remains severe [56]. In another study, the complete inclusion of a pseudo-exon due to the c.3469−1304C>G variant has been clearly associated with the most severe phenotypes compared with the c.2989−313A>T and c.3874−4522A>G variants [47]. The absence of the c.2989−313A>T and c.3874−4522A>G deep intronic variants in healthy individuals suggests their full penetrance, although they have been associated with some very mild phenotypes (i.e., phenotypic variability) [47].

The combination of mRNA analysis and in vitro splicing studies can be used to determine the level of full-length *CFTR* transcripts [25] and the phenotypic spectrum associated with the variant. Variability in the endogenous normal mRNA rate may also depend on the NMD activity (not triggered in intronless minigene assays), on inter-individual variation in gene expression (expression of trans-acting splicing regulatory factors) or on non-genetic factors (infections, environment). Studies reported that low levels (5–10%) of normal *CFTR* mRNA ameliorate the severity of pulmonary disease in CF [22,57–60].

Further understanding of the amount of correctly spliced mRNA will contribute to define the eligibility for existing therapies of patients carrying splicing mutations or to develop new targeted splicing modulators.

## Splicing mutations and therapeutics

The importance of splicing mutations in the pathogenesis of genetic diseases has led to the development of drugs that can reverse their effect. One of the challenges in designing such therapeutics is ensuring their specificity.

### Small-molecules splicing modulators

Several small molecules that can recognize specific three-dimensional structures have been developed. For example, in myotonic dystrophy, an autosomal dominant genetic disorder, the expanded repeats ((CTG)n repeat expansion in the 3′ untranslated region of the *DMPK* gene for DM1 and (CCTG)n repeat expansion in intron 1 of the *ZNF9* gene for DM2) are toxic as the repeats can form a stable structured RNA that aberrantly sequesters with proteins such as MBNL1 in the nucleus. Sequestration of MBNL1, an alternative splicing factor, leads to the missplicing of multiple pre-mRNAs in myotonic dystrophy. Two small molecules, pentamidine and Hoechst 33258 modulate alternative splicing in myotonic dystrophy by disrupting binding of the alternative splicing factor MBNL1 to expanded CUG in vitro and in vivo [61, 62]. Risdiplam [63] is a splicing modulator that increases exon 7 inclusion in the *SMN2* gene, thereby increasing the levels of SMN protein throughout the organism. This brain-penetrant oral drug has been evaluated in patients with spinal muscular atrophy (SMA) with promising results [64].

Spliceosome-mediated RNA trans-splicing (SMaRT) is an alternative strategy in which RNA trans-splicing molecules are used to replace the entire coding sequence upstream or downstream of a target splicing site to correct aberrant mRNAs. This approach has been evaluated in CF [65] and in other diseases, such as hemophilia A, retinitis pigmentosa and SMA [66–68].

Strategies to precisely control gene replacement or gene editing by exposure to small-molecule inducers also have been developed [69]. The small-molecule inducers currently tested for human use take advantage of alternative RNA splicing. This strategy was first developed for the treatment of SMA by using drugs to influence exon skipping in an *SMN2* minigene and obtain in-frame gene expression. This approach is used for exons in which splicing is responsive to therapeutic agents. Experiments in mice showed that the exon splicing of the injected minigene was induced in a dose-dependent manner by oral administration of the drugs [69].

### Oligonucleotide-based therapies

The success of two therapies for spinal muscular atrophy and Duchenne muscular dystrophy opened the path to antisense oligonucleotide (AON)-based therapies. AONs are short synthetic DNA or RNA molecules that target pre-mRNA fragments and can modulate the splicing process. Specifically, they can prevent aberrant splicing or induce additional exon exclusion to restore the proper reading frame. AON-based therapy can be directed against a broad variety of targets, also those that are considered undruggable at the protein level. When the genetic information is known, AON design is a rational, fast and efficient process.

Several AON-based therapies are now marketed in the United States and Europe, such as fomivirsen (the first approved by the US FDA for cytomegalovirus retinitis), mipomirsen for familial hypercholesterolemia (it binds to APOB-100 transcripts and induces RNase H-mediated cleavage of the targeted RNA), and eteplirsen (binds to exon 51 of the *DMD* gene to produce partially functional dystrophin protein) and more recently givosiran and golodirsen (they bind to exon 53 of *DMD*) for Duchenne muscular dystrophy (for review: [70]). In 2016, Spinraza^TM^ was approved by the FDA for the treatment of SMA. This AON targets the intron 7 internal splice site within the *SMN2* pre-mRNA to promote exon 7 inclusion and thus to produce a functional SMN protein [71].

Oligonucleotides are chemically modified to improve their efficacy, limit off-target side effects, and avoid target pre-mRNA degradation in the AON/pre-mRNA duplex by RNase H. A significant progress in this field has been the identification of novel AON modifications [72], such as changes in the phosphate backbone or nucleobase or sugar rings, to improve their enzymatic stability, aqueous solubility, binding affinity and cell uptake, and to limit immune stimulation [73]. AONs usually have a phosphorothioate (PS) backbone [74]. Ribose sugar can contain 2′-O-methyl or 2′-O-methoxyethyl substitutions that increase the oligonucleotide stability [75]. AON modifications at sugar positions include also phosphorodiamidate morpholino, peptide nucleic acids, and Locked Nucleic Acids (LNA) that increases binding affinity to the target and resistance to nucleases [76]. LNA are artificial analogs in which the ribose contains a bridge connecting the 2′-O with the 4′-C position. Oligonucleotides are often designed as LNA/DNA mixmers (oligonucleotides with alternating LNA and DNA residues). In this AON class, miravirsen (anti-miR-122) is the first anti-miR LNA that efficiently inhibits miR-122, resulting in a prolonged and dose-dependent decrease in the RNA levels of hepatitis C virus (single-stranded RNA virus) (ongoing trial) [77]. MRG-106 (anti-miR-155) is another LNA that targets miR-155 for the therapy of cutaneous T cell lymphoma [78]. In the case of CF, we used LNA/PS-modified oligonucleotides to target the 3′-UTR of *CFTR* mRNA with the aim of preventing binding of miR-101 and miR-145 that are overexpressed in CF [79, 80]. The transfection of these molecules in bronchial epithelial cells of patients with CF leads to an increase of CFTR expression and activity [79]. AON strategy was recently applied to promote exon skipping of exon 23 to eliminate the W1282X nonsense mutation and to avoid RNA degradation induced by the NMD mechanism [81–83].

In addition to AON, various technologies are in development to harness the approaches. Bifunctional oligonucleotides are made of an antisense portion targeting a specific sequence and a non-hybridizing tail that recruits acting factors to modulate splicing outcome, silencing (TOSS, targeted oligonucleotide silencer of splicing) or enhancing (TOES, targeted oligonucleotide enhancer of splicing) [84, 85].

### AON-based therapies for CF splicing mutations

AON have been explored previously as a tool to correct defective splicing on CF. This strategy has been successively tested to correct the aberrant splicing caused by the CFTR c.2657+5G>A splicing mutation (2789+5G>A), leading to exon 16 skipping, in cell lines with minigene constructs [86]. Two AON have been designed to target either downstream (AON1) or upstream (AON2) to the U1 snRNA binding region. AON1 transfection almost completely corrected the exon 16 inclusion, possibly by masking a binding site for an inhibitor splicing regulator (ISS). Introduction of AON2 showed lower efficiency, possibly it slightly impairs correct exon recognition [86]. AON-based strategies are particularly suitable for deep intronic mutations because these sequences are far away from exons, and therefore the AON will not interfere with the regulatory motifs located near exons for the recruitment of trans-regulatory proteins that participate in the inclusion choice. The ability of modified AON (2′-*O*-methyl/PS) to reduce the aberrant splicing induced by the c.3718−2477C>T intronic mutation has been demonstrated over twenty years ago [87]. More recently, two other studies demonstrated that treatment of patient-derived cells with AON directed against the aberrant splicing induced by the c.3718−2477C>T mutation is effective, underlining the need to optimize AON design. Screening of 26 consecutive AON targeting the c.3718−2477T allele, along the entire 84 bp cryptic exon and its 50 bp flanking intronic sequences led to the selection of one candidate that induced a full rescue of CFTR function in primary respiratory epithelial cells derived from patients homozygous for the c3718-2477T mutation [88, 89]. Functional analysis was also performed on several primary respiratory epithelial cells derived from patients heterozygous for that mutation, in which the response of the CFTR activity was of 43% of WT, 50% being the maximum possible effect in heterozygotes [88]. More recently, it was reported that the 2′-*O*-methyl/PS-based AON improves by 3-fold the CFTR chloride channel currents (versus 2-fold by VX770/VX809) in CF patient-derived nasal and bronchial epithelial cells (p.Phe508del/c.3718-2477T) [88, 89]. We used a similar approach in which LNA/PS-based AONs target the aberrant splicing generated by two other intronic CFTR mutations, the c.1680−883A>G and c.1680−886A>G. AONs designed to block access to the 3′ acceptor splice site successfully restored normal splicing with a strong corrective effect (up to 70% of CFTR expression) at low concentration (50 nmol/l) for the both mutations in cell lines and in primary nasal cultures [12]. Some patients with intronic splicing mutations in trans to other CF mutations are now eligible for currently available CFTR modulators including patients carrying the 3849+10kbC>T [90] (Supplementary Tables 1 and 2). However, a limited clinical benefit of CFTR modulators for patients carrying this mutation has been observed and no additional effect of VX-770 has been showed when added to AON candidates [88]. These data underline that patients with splicing CFTR mutations could greatly benefit from the development of a similar AON-based strategy.

### Genome-editing approaches in CF

New molecular biology and genetic technologies have led to the development of targeted genome editing approaches using the clustered regularly interspaced short palindromic repeats-Cas9 (CRISPR-Cas9) system. Recent data showed that CRISPR/Cas9-based targeted excision of short intronic sequences containing mutations that create cryptic splice signals can restore normal splicing in a *CFTR* hybrid minigene [91, 92]. Another study reported that the splicing defect induced by the c.3718−2477C>T (3849+10kbC>T) and c.3140−26A>G CFTR mutations can be efficiently and precisely corrected using the AsCas12a nuclease in CF patient-derived intestinal organoids [93], thus offering a new alternative for CF therapy.

## Summary

Mutations affecting the splicing pattern can cause genetic disorders, and their frequency might have been underestimated. In CF, some splicing mutations have been erroneously classified as synonymous changes or benign amino acid substitutions. Particularly, the frequency and impact of deep intronic mutations have been undervalued, and the development of therapeutics to target splicing mutations neglected. The implementation of new technologies gave the opportunity to design/develop new drugs that can reverse the effect of splicing mutations. Although the administration mode and delivery systems that influence drug bioavailability and efficiency remain challenging, the promising results of the AON-based splice-switching therapeutics in ongoing clinical trials demonstrate that the treatment of genetic disorders caused by splicing defects is possible and offer new hopes for personalized therapies in CF.

## Supplementary information

Supplementary Table 1

Supplementary Table 2
