# Supplementary material for: Splicing mutations in the CFTR gene as therapeutic targets
Source: Gene Ther. 2022 Jun 2;29(7-8):399–406. doi: 10.1038/s41434-022-00347-0 (PMC9385490; doi:10.1038/s41434-022-00347-0)
Supplement: Supplementary file 1 — Supplementary Table 1 [file 41434_2022_347_MOESM1_ESM.pdf]

Supplementary Table 1 – Variants located at canonical splice site or at closed position described to affect exon inclusion (CFTR2, https://cftr2.org).

Variant cDNA name *(HGVS-nomenclature)*	Variant legacy name	Intron	Allele frequency in CFTR2 (of 142,036 identified variants)	% pancreatic insufficient	Variant final determination 24 september 2021 (current version)	Eligible to
**c.53+1G>T**	185+1G->T	1	0.00006	100%	CF-causing	
**c.164+1G>A**	296+1G->A	2	0.00002	100%	CF-causing	
**c.164+1G>T**	296+1G->T	2	0.00003	100%	CF-causing	
**c.164+2T>C**	296+2T->C	2	0.00002	100%	CF-causing	
**c.164+4dupT**	296+3insT	2	0.00003	50%	CF-causing	
**c.165-1G>A**	297-1G->A	2	0.00004	100%	CF-causing	
**c.273+1G>A**	405+1G->A	3	0.00027	100%	CF-causing	
**c.273+3A>C**	405+3A->C	3	0.00007	100%	CF-causing	
**c.274-2A>G**	406-2A->G	3	0.00004	100%	CF-causing	
**c.274-1G>A**	406-1G->A	3	0.00028	100%	CF-causing	
**c.489+1G>T**	621+1G->T	4	0.00931	99%	CF-causing	
**c.489+3A>G**	621+3A->G	4	0.00007	25%	VCC	Kalydeco (in trans with p.Phe508del)
**c.579+1G>T**	711+1G->T	5	0.00193	98%	CF-causing	
**c.579+3A>G**	711+3A->G	5	0.00044	21%	CF-causing	Kalydeco/Symdeco
**c.579+5G>A**	711+5G->A	5	0.00042	83%	CF-causing	
**c.580-2A>G**	712-2A->G	5	0,00002	100%	CF-causing	
**c.580-1G>T**	712-1G->T	5	0.00030	100%	CF-causing	
**c.744-2A>G**	876-2A->G	6	0,00002	100%	CF-causing	
**c.744-6T>G**		6				
**c.1116+1G>A**	1248+1G->A	8	0.00020	100%	CF-causing	
**c.1117-1G>A**	1249-1G->A	8	0.00004	100%	CF-causing	
**c.1209+1G>A**	1341+1G->A	9	0.00008	86%	CF-causing	
**c.1210-2A>C**	1342-2A->C	9	0,00006	67%	CF-causing	
**c.1393-1G>A**	1525-1G->A	10	0.00051	100%	CF-causing	
**c.1393-2A>G**	1525-2A->G	10	0.00006	100%	CF-causing	
**c.1584+1G>A**	1716+1G->A	11	0.00004	50%	CF-causing	
**c.1585-1G>A**	1717-1G->A	11	0.00856	97%	CF-causing	
**c.1585-8G>A**	1717-8G>A	11	0,00019	100%	CF-causing	
**c.1679+1G>C**	1811+1G->C	12	0.00012	92%	CF-causing	
**c.1679+1G>A**	1811+1G->A	12	0.00004	100%	CF-causing	
**c.1680-1G>A**	1812-1G->A	12	0.00023	100%	CF-causing	
**c.1766+1G>A**	1898+1G->A	13	0.00296	99%	CF-causing	
**c.1766+1G>C**	1898+1G->C	13	0.00004	100%	CF-causing	
**c.1766+1G>T**	1898+1G->T	13	0.00005	100%	CF-causing	
**c.1766+3A>G**	1898+3A->G	13	0.00019	28%	CF-causing	
**c.1766+5G>T**	1898+5G->A	13	0,00004	80%	CF-causing	
**c.2490+1G>A**	2622+1G->A	14	0.00060	98%	CF-causing	
**c.2657+5G>A**	2789+5G>A	16	0.00723	43%	CF-causing	Kalydeco/Symdeco
**c.2658-1G>C**	2790-1G->C	16	0.00005	100%	CF-causing	
**c.2988+1G>A**	3120+1G->A	18	0.00353	98%	CF-causing	
**c.2989-1G>A**	3121-1G->A	18	0.00014	100%	CF-causing	
**c.2989-2A>G**	3121-2A->G	18	0.00005	100%	CF-causing	
**c.3368-2A>G**	3500-2A->G	20	0.00009	88%	CF-causing	
**c.3468+2dupT**	3600+2insT	21	0,00008	100%	CF-causing	
**c.3468+5G>A**	3600+5G->A	21	0,00002	33%	CF-causing	
**c.3717+4A>G**	3849+4A->G	22	0.00010	22%	CF-causing	
**c.3717+5G>A**	3849+5G->A	22	0.00002	100%	CF-causing	
**c.3718-1G>A**	3850-1G->A	22	0.00008	91%	CF-causing	
**c.3718-3T>G**	3850-3T->G	22	0,00004	100%	CF-causing	
**c.3873+1G>A**	4005+1G->A	23	0.00015	100%	CF-causing	
**c.3873+2T>C**	4005+2T->C	23	0.00011	27%	CF-causing	
**c.4242+1G>T**	4374+1G->T	26	0.00011	93%	CF-causing	
**c.4242+1G>A**	4374+1G->A	26	0.00006	100%	CF-causing	

VCC (Varying clinical consequence): when in trans with another CF-causing mutation, variants can either result in CF or in a CFTR-RD
