# Supplementary material for: Splicing mutations in the CFTR gene as therapeutic targets
Source: Gene Ther. 2022 Jun 2;29(7-8):399–406. doi: 10.1038/s41434-022-00347-0 (PMC9385490; doi:10.1038/s41434-022-00347-0)
Supplement: Supplementary file 2 — Supplementary Table 2 [file 41434_2022_347_MOESM2_ESM.pdf]

Supplementary Table 2 - Disease-causing deep intronic variants in the *CFTR* gene

Variant cDNA name	Variant legacy name	Intron	AS/DS (exon cryptic length)	Number of patients carrying this variant in		Variant final determination in		References	Eligible to
				CFTR2	CFTR-France	CFTR2	CFTR-France		
**c.870-1113-870-1110del**	870-1113-870-1110delGAAT	7	ISE creation (101 nu)	NR	21 CF<br>8 CFTR-RD	NR	Disease-causing (VCC)	(43)	
**c.1585-9412A>G**	c.1584+18672A>G	11	DS<br>(104bp and 65 nu)	NR	5 CF	NR	CF-causing	(44)	
**c.1680-877G>T**	1811+1643G->T	12	DS<br>(53 nu)	22	NR	CF-causing	NR	(45)	
**c.1680-886A>G**	1811+1.6kbA>G	12	DS<br>(49 nu)	75	27 CF	CF-causing	CF-causing	(46)	
**c.1680-883A>G**	-	12	DS<br>(53 nu)	NR	3 CF	NR	CF-causing	(12)	
**c.2989-313A>T**	-	18	DS<br>(118 nu)	NR	3 CF<br>1 CFTR-RD	NR	Disease-causing (VCC)	(12,47)	
**c.3469-1304C>G**	3600 + 11.5kbC>G	21	DS<br>(214 nu)	NR	5 CF	NR	CF-causing	(48)	
**c.3717+40A>G**	3849+40A>G	22	DS<br>(40nu)	13	4 CF<br>3 CFTR-RD	CF-causing	Disease-causing (VCC)	(49)	
**c.3718-2477C>T**	3849+10kbC>T	22	DS<br>(84 nu)	1,102	43 CF<br>11 CFTR-RD	CF-causing	Disease-causing (VCC)	(22,50,51)	Kalydeco/Symdeco<br>Trikafta (in trans with p.Phe508del)
**c.3874-4522A>G**	4005 + 5727A>G	23	AS<br>(125 nu)	NR	4 CF<br>1 CFTR-RD	CF-causing	Disease-causing (VCC)	(12,47,52)	Trikafta (in trans with p.Phe508del)

ISE: Intronic Splicing Enhancer; DS: Donor Site; nu: nucleotides; NR: not referenced; CF: patients with Cystic Fibrosis; CFTR-RD: patients with CFTR-related disorders including CBAVD (Congenital bilateral absence of the vas deferens, a cause of male infertility), bronchectasis, pancreatitis…; CF-causing: when in trans with another CF-causing mutation, variants will result in CF; VCC (Varying clinical consequence): when in trans with another CF-causing mutation, variants can either result in CF or in a CFTR-RD.
